# Advances in Nursery Production of Hazelnut Plants in Serbia − Successful Grafting of Different *Corylus avellana* L. Cultivars and Clones Onto *Corylus colurna* L. Rootstock

**DOI:** 10.3389/fpls.2021.785015

**Published:** 2021-12-17

**Authors:** Sandra Bijelić, Nenad Magazin, Sava Džankić, Draža Janković, Borivoje Bogdanović, Goran Jaćimović

**Affiliations:** ^1^Department for Fruitgrowing, Viticulture, Horticulture and Landscape Architecture, Faculty of Agriculture, University of Novi Sad, Novi Sad, Serbia; ^2^OZZ Leska Nursery, Dobrić, Serbia; ^3^Department for Field Crops and Vegetables, Faculty of Agriculture, University of Novi Sad, Novi Sad, Serbia

**Keywords:** hazelnut, Corylus, Turkish filbert, grafting, cultivar, clone, rootstock

## Abstract

The latest trends in hazelnut production are moving in the direction of selection and breeding of more productive cultivars, isolation of native clones, and more intensive clonal selection of rootstocks aimed at enhancing the agronomic performance of plants. Serbia stands out in the production of quality planting material by grafting on Turkish filbert (*Corylus colurna* L.), which does not form shoots and develops in the form of a tree. The aim of this research was to investigate the success achieved by grafting leading Italian cultivars (Tonda gentile romana, Tonda di Giffoni, and Tonda Gentile della Langhe) and their clones on Turkish filbert seedlings using technology developed at the University of Novi Sad, Faculty of Agriculture, Serbia, as well as determine possible differences in the quality and variability of the obtained planting material. For this purpose, from the end of March to the end of April, two-year-old *C. colurna* generative rootstocks (seedlings) were grafted by the whip and tongue method. At the beginning of September, the grafted plants were counted, and after the plants entered the dormant period (autumn in the year of grafting), they were taken out of the soil and classified. The obtained results revealed that the chosen hazel cultivars and clones exhibited excellent grafting success rate. In both analyzed years, as well as throughout the entire study period, greater grafting success was achieved using clones relative to the main cultivars. Over the two-year study period, the highest grafting success was achieved by clone AD17. Class I grafted plants were obtained in 80% of the cases, especially with Tombesi and AD17 clones, while significantly fewer Class I grafted plants were produced by grafting basic cultivars. Clones AD17 and Tombesi also produced grafted plants of greatest height and graft union diameter. All clones exhibited superior uniformity (i.e., a more stable grafting success) relative to their basic cultivars.

## Introduction

The hazelnut industry is becoming increasingly focused on the selection and breeding of more productive cultivars, isolation of native clones and more intensive clonal selection of rootstocks as a means of improving the agronomic output. In the case of hazelnuts, this trend is even more specific, since the production of this nut crop is tailored to the agroecological conditions, both in terms of cultivar selection and cultivation technology. In the areas known for hazelnut production, shrubs are typically grown, and rooted suckers serve as the most common propagation material, despite limited sanitary control associated with this approach. However, ample body of evidence shows that nursery techniques, such as layering (Lagerstedt, 1983; Solar et al., 1994), softwood and hardwood cuttings (Kantarci and Ayfer, 1994; Ughini and Roversi, 2005), *in vitro* propagation (Damiano et al., 2005), and grafting are more suitable for use in the modern hazelnut industry. In perennial fruit crops in particular, grafting has been used for millennia for vegetative propagation, as it can improve some agronomic characteristics, such as yield or vigor, as well as tolerance to biotic and abiotic stresses (Loupit and Cookson, 2020). In Serbia, *Corylus avellana* L. cultivars are typically grafted on the *Corylus colurna* L. (Turkish filbert) rootstock, as this technology that has been developed and improved in this country results in high-quality planting material. Mastering the technology of grafting hazelnut cultivars onto Turkish filbert and mass production of grafted hazelnut plants began at the end of the 20th century at the Faculty of Agriculture in Novi Sad (Ninić Todorović and Antanasović, 1989; Ninić Todorović et al., 1994, 1995a,b; Korać et al., 1996a,b; Cerović et al., 1998).

The fact that grafted hazelnuts are a good source of planting material is also indicated by the growing interest of producers from many countries in raising them. The first plantations of grafted hazelnuts with planting material from Serbia were erected in Italy and France, with the intention of further expansion, and there is interest from Chile, Russia, Belarus, Croatia, and other countries (Cerović et al., 2020). According to Rovira (2021), hazelnut plantations are increasingly being established using grafted hazel plants, primarily on *C. colurna* L., since selected clones of other rootstocks have been shown to form shoots on trees over time. In addition, the use of different scion/rootstock combinations may be associated with grafting incompatibility, which generally increases with taxonomic distance (Goldschmidt, 2014). On the other hand, many years of research and selection of *C. colurna* L. genotypes offer ample evidence that it is the optimal rootstock for hazelnut cultivars (Ninić Todorović, 1990). In Serbia, hazel cultivar grafting on the Turkish filbert rootstocks has been successfully performed since 1989, as reported by Ninić Todorović (1990), Ninić Todorović et al. (1994, 2003, 2006, 2007, 2008, 2012), Korać et al. (1993, 1994a,b), Cerović et al. (2007), and other authors. In Serbia, hazel is grafted exclusively on Turkish filbert, which does not produce shoots but rather develops in the form of a small tree, which can live up to 200 years, and has a good affinity with *C. avellana* cultivars. As Turkish filbert has a thick cork bark, unlike other fruit species grafted by budding, it is exclusively grafted by a one-year lignified shoot with one or two internodes. As a result, it is necessary to produce quality rootstock as well as graftwood.

Modern hazelnut cultivation implies full application of mechanization, which is only possible if the hazelnut is grown in the form of a small tree, which can be ensured by grafting or by destroying the root shoots. According to Radicati et al. (1994), the formation of *C. avellana* shoots depends on the cultivar., climate, soil, propagation technique, and cultivation technology. Therefore, their control is one of the most important hazelnut planting management operations, unless plants grafted on rootstocks that do not produce shoots are used (Cerović et al., 2008). In hazelnut orchards, the suckers must be eliminated (by desuckering) at least twice per year. Currently, desuckering is performed by various means, including manually, mechanically, by using desiccants, and through application of flame (Tomasone et al., 2009). Each of these methods is both labor and time-intensive as well as expensive, and typically requires at least one repetition. In addition, desiccants are not allowed in organic farming (Roversi, 2015). To overcome these issues, intensive work is being done on the evaluation of non-suckering rootstocks. Rovira et al. (2014) noted that *C. avellana* non-suckering clonal rootstock “MB-69” and *C. colurna* clonal rootstocks “Dundee” and “Newberg” improve the agronomic performance of “Negret N·9” cultivar. However, Roversi (2015) is of view that the best (albeit relatively recent) alternative that is successfully applied only in Serbia, is the grafting of *C. avellana* cultivars on *C. colurna* seedling. In recent decades, Italy has significantly improved hazelnut production, primarily by creating hazelnut plants *in vitro* and selecting leading cultivars, as well as by isolating native clones for use in mass production. Biancolillo et al. (2018) developed a non-destructive method for the authentication of a specific high-quality Italian hazelnut Nocciola Romana, registered with a protected designation of origin (PDO). Cultivars that are recommended for use in Italy due to their superior nut quality, productivity, vigor and type of growth are Tonda di Giffoni, Tonda Gentile delle Langhe, and Tonda Romana (Carvajal Rodriguez et al., 2018). In Italy, clonal hazel selection began in the 1960s, focusing on Tonda Gentile delle Langhe (Romisondo et al., 1983; Valentini et al., 2001), Tonda gentile romana (Monastra et al., 1997), and Tonda di Giffoni (Limongelli, 1983; Limongelli and Piccirillo, 2002).

Earlier research suggests that clonal selection has yielded modest results in terms of improving fruit quality. Romisondo et al. (1983) found no differences among clones selected from the Tonda Gentile delle Langhe population, but these results were countered more recently by Valentini et al. (2001), and small variations were found among clones selected from the Tonda gentile romana population by Monastra et al. (1997). Clone selection was rated by Andreakis et al. (2002) as less efficient in species propagated by shoots compared to those propagated by grafting. Recent investigation of genetic diversity of Turkish *C. avellana* hazelnut cultivars conducted by Oztolan-Erol et al. (2021) revealed presence of high intra-cultivar diversity, while also indicating that several cultivars were genetically admixed. These authors also identified high genetic diversity within the cultivar itself. Thus, recently published results and clone descriptions confirm the importance of clonal selection, whereby most successful clones are produced in Italy and are thus recommended for use in cultivation (Petriccione et al., 2010; Valentini et al., 2014).

Considering that technology for the production of grafted hazelnut planting material which does not produce suckers has been developed in Serbia, and since grafting allows for fast multiplication of new cultivars and clones, the nursery OZZ “Leska” from Serbia was given the permission to multiply the most sought-after clones of the leading Italian hazelnut cultivars. The aim of this research was to examine the success of this initiative, as a part of which recommended Italian cultivars and their clones were grafted on Turkish filbert seedling, using the technology developed at the Faculty of Agriculture in Novi Sad. Its further goal was to determine possible differences in the quality and variability of planting material between selected cultivars and clones.

## Materials and Methods

### Site Description and Plant Material

The present study spanned a two-year period (2020 and 2021) and was conducted in the nursery of OZZ “Leska” located in the Dobrić village (44° 42′ 04″ N; 19° 34′ 32″ E, 90 m a.s.l.) belonging to the Šabac municipality in western Serbia (Mačva region). The soil where the trial was set up is flat, medium-deep Pseudogley according to the Serbian soil classification system, while according to WRB classification Food and Agriculture Organization of the United Nations (FAO) (2014) this soil type is classified as Planosol. The surface horizons (A and g) are silt loam, with 40% silt, while the impermeable layer is clayey loam with a greater clay content than in the surface horizons. The humus horizon has high porosity (50%), and its air capacity is 15%. However, in the g horizon, these values decrease, as soil contains 2–3% humus in analyzed fields, but this percentage declines with depth. Nursery OZZ “Leska” is the only nursery in Serbia that is registered for copyright protection and reproduction of tested hazel clones. It has been engaged in nursery production for almost three decades and specializes in the production of hazelnut planting material by grafting on the Turkish filbert rootstock. For the present study, two-year-old generative rootstocks (seedlings) of Turkish filbert were produced according to the standard procedure (Ninić Todorović et al., 1994; Cerović et al., 2020). These non-clonal rootstocks were grafted by the “whip and tongue” method in the period from the end of March to the end of April (early spring) during both years. The graftwood was taken from the propagation stock of both cultivars and clones. Three basic cultivars were grafted, namely Tonda gentile romana (TGR), Tonda di Giffoni (TDG), and tonda gentile della langhe (TGDL), as well as their clones, denoted as TGR (Clone 1 and Clone 3), TDG (Tombesi), and TGDL (AD17 and PD6). Tonda di Giffoni is a highly valued cultivar due to its productivity, kernel blanching, rapid growth, medium vigor, a remarkable protandry and self-incompatibility, as well as early female and male flowering (Tombesi and Limongelli, 2002; Grau, 2003; Ellena et al., 2014). On the other hand, Tonda Gentile delle Langhe, which originated in Piedmont, northern Italy, has remarkable protandry and self-sterility, as well as habit of both intermediate and moderate vigor (Ellena et al., 2014). Finally, Tonda Romana is characterized by a medium-low vigor, late budbreak, medium productivity and medium-late maturing (Tombesi and Limongelli, 2002; Grau, 2009). After grafting, standard measures of care and protection were applied in the nursery during the vegetation phase. At the beginning of September, grafted plants were counted, their height and diameter just above graft union were measured, and after leaf fall, once the plants entered the dormant period (in autumn in the year of grafting), plants were pulled out of the soil and classified according to the Rulebook on quality standards, packaging, sealing and declaration of planting material of agricultural plants in Serbia. Defined quality standards for Class I plants are as follows: at least five branch roots of 0.2 m length each, 0.7 m plant length above the graft union, and 10 mm diameter just above the graft union. Provided that they received adequate care, hazel plants are ready for planting in the fall of the same year in which they were grafted. Taking into account the time needed to produce the rootstock, the production of grafted hazel plants takes three vegetation periods.

### Weather Conditions in the Nursery Throughout the Experiment

**Figure 1:**
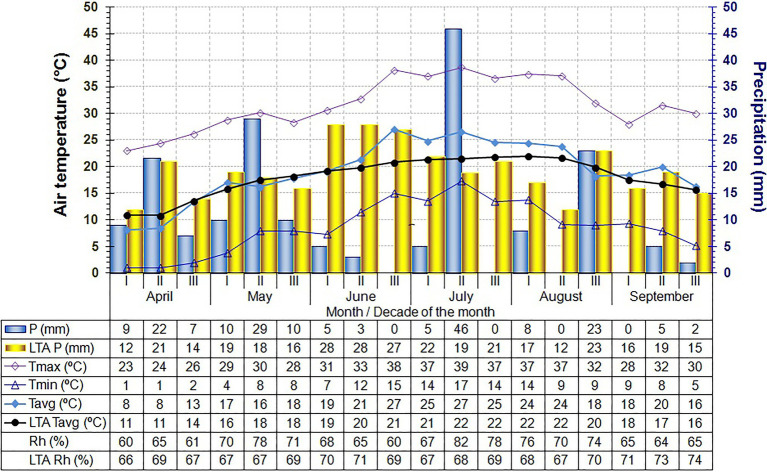
Main climatic parameters in the nursery throughout the experiment (April–September, by decades of each month) in 2020. P (mm), Precipitation sum (mm); Tmin (°C), Minimum air temperature (°C); Tmax (°C), Maximum air temperature (°C); Tavg (°C), Average air temperature (°C); Rh (%), Relative air humidity (%); LTA, Long-term average for analyzed climate parameters.

**Figure 2:**
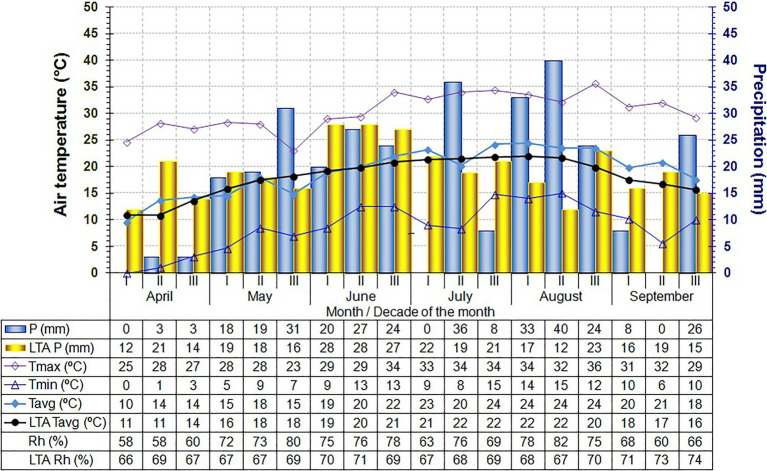
Main climatic parameters in the nursery throughout the experiment (April–September, by decades of each month) in 2021.

The climatic conditions at the locality have all the characteristics of a temperate continental climate. Weather conditions (temperature, precipitation, and air relative humidity) for the April–September period in 2020 and 2021 were monitored by the automatic weather station Šabac (44° 75′ N, 19° 69′ E, 79 m a.s.l.), and the weather data are shown in Figures 1, 2.

### Statistical Analysis

The data set was tested in line with the basic assumptions of ANOVA. One of the assumptions that must be met for ANOVA application is normal data distribution with a common variance. This assumption can be violated if the data set is heterogeneous, especially when values are reported as percentages. To overcome this issue, the data needs to be transformed to achieve normal distribution. For this reason, the Shapiro–Wilk W test was conducted in the present study for evaluating normality of distributions related to all measured variables. The null hypothesis of this test is that the data are normally distributed. If the W statistic is significant (at *α* = 0.05), the hypothesis that the evaluated distribution is normal should be rejected. In our cases, for Success of grafting, the W value was 0.97023 with *p* = 0.05872, which was greater than 0.05, indicating that the data were normally distributed. For the percentage of Class I grafted plants, we obtained *W* = 0.96899 and *p* = 0.04896, which was below the chosen alpha level, indicating that data were not normally distributed. Consequently, the *arcsine* (angular) method was employed to transform the percentages, in line with the approach described by Gomez and Gomez (2010), and the transformed data were subjected to parametric ANOVA (however, untransformed means were reported in relevant tables). When examining the agronomic performance of grafted plants for normality of distribution, the Shapiro–Wilk test yielded *W* = 0.99141 and *p* = 0.87644 for height, and *W* = 0.99267 and *p* = 0.93373 for the diameter, confirming that both data sets were normally distributed.

The ANOVA procedure was performed according to a completely randomized design with four replicates (1,000 plants were analyzed per one replication). Factorial ANOVA procedure was performed considering Years (Y) and Cultivars and their Clones (C) as fixed treatments, with 95% confidence interval. The statistical significance of the difference among the means of *percentage* of graft success and of Class I grafted plants was determined using Fisher’s least significant difference (LSD) *post hoc* test at the 5% probability level. For testing significance of the difference among the means of *measured* agronomic performance (height and diameter) of grafted plants, we used Duncan’s multiple range test at the same probability level. ANOVA analysis was performed using the TIBCO Statistica software package, version 13.3.0 (TIBCO Software Inc., Palo Alto, CA, United States).

## Results

According to the ANOVA results shown in Table 1, only the main factor C (Cultivars/Clones) exerted significant effects (probability value = 0.044; bolded) on the total variability in the grafting success, whereas Year and Y × C interactions were not significant (Pr = 0.273 and 0.969, respectively). As indicated in Table 2, the effect of treatments on the percentage of young plants of Class I was not statistically significant. However, due to the relatively low probability value of treatment C (Pr = 0.101), the percentage of Class I young plants was also subjected to a *post hoc* Fisher’s LSD test.

**Table 1:** ANOVA results related to grafting success^*^.

Source	d.f.	SS	MS	*F*	Pr > *F*
Model	19	4431.715	233.248	1.203	0.286
Y	1	237.763	237.763	1.226	0.273
C	9	3654.999	406.111	2.095	0.044
Y × C	9	538.953	59.884	0.309	0.969
Error	60	11631.887	193.865	–	–
Total	79	16063.601	–	–	–

* *ANOVA, Analysis of variance involving basic parameters: d.f., Degrees of freedom; SS, Sum of squares; MS, Mean squares; F, variance ratio (F test); Pr, Probability value corresponding to a variance ratio*.

**Table 2:** ANOVA results related to the percentage of Class I grafted plants^*^.

Source	d.f.	SS	MS	*F*	Pr > *F*
Model	19	2138.213	112.538	1.170	0.312
Y	1	227.171	227.171	2.362	0.130
C	9	1501.636	166.848	1.735	0.101
Y × C	9	409.407	45.490	0.473	0.887
Error	60	5771.201	96.187	–	–
Total	79	7909.414	–	–	–

* *ANOVA, Analysis of variance involving basic parameters: d.f., Degrees of freedom; SS, Sum of squares; MS, Mean squares; F, variance ratio (F test); Pr, Probability value corresponding to a variance ratio*.

### Success of Grafting

Grafting success is one of the main indicators of the viability of propagation methods, including those adopted in our trial. As can be seen from the results reported in Table 3, the chosen hazel cultivars and clones exhibit excellent grafting success rate. The highest grafting success rate was recorded in 2020 for clone AD17 (85.80%); however, this result was significant only in relation to the cultivar TGR with the lowest grafting success (65.50%). No significant difference in grafting success was observed between other examined cultivars and clones, and it ranged from 69.40% (clone PD6) to 84.10% (clone Tombesi). Clone AD17 also had the best reception (85.23%) in the second study year (2021), which was significantly higher than that recorded for the cultivars TGDL (61.30%), TGR (63.56%) and Clone 1 (64.40%). Moreover, clones Tombesi and MT5 had significantly higher graft success compared to the TGDL cultivar. No significant differences in graft success were observed between other examined cultivars and clones, and their values ranged from 72.99% (clone PD6) to 79.36% (clone MT4), as shown in Table 3. It is worth noting that, in both analyzed years, with the exception of clone PD6, all clones had a higher grafting success in relation to their basic cultivars. In the case of PD6, the basic cultivar TGDL had a greater (albeit statistically insignificantly) success.

**Table 3:** Percentage of grafting success and Class I young plants within two years (Y) and ten cultivars and their clones (C) and the corresponding coefficient of variation (CV)^*^.

Year	Cultivars/Clones	Grafting Success (%)	CV (%)	Young plants of Class I (%)	CV (%)
2020	TGDL	74.18 ± 15.04 a–e	20.3	73 ± 9 cd	12.3
	Clone MT4	83.86 ± 13.93 a–c	16.6	78 ± 12 a–d	15.3
	Clone MT5	80.70 ± 14.34 a–e	17.8	74 ± 7 b–d	9.0
	Clone AD17	85.80 ± 14.14 a	16.5	84 ± 10 a–c	12.0
	Clone PD6	69.40 ± 12.82 a–e	18.5	80 ± 10 a–d	12.5
	TGR	65.50 ± 14.72 b–e	22.5	76 ± 10 b-d	13.7
	Clone 1	76.85 ± 12.02 a–e	15.6	75 ± 7 b–d	9.5
	Clone 3	73.94 ± 12.89 a–e	17.4	81 ± 9 a–d	10.9
	TDG	78.24 ± 15.25 a–e	19.5	78 ± 11 a–d	13.6
	Clone TOMBESI	84.10 ± 13.93 ab	16.6	85 ± 6 a–c	7.0
2021	TGDL	61.30 ± 13.55 e	22.1	69 ± 10 d	14.4
	Clone MT4	79.36 ± 13.62 a–e	17.2	81 ± 10 a–d	12.3
	Clone MT5	81.56 ± 14.02 a–d	17.2	86 ± 10 a–c	11.3
	Clone AD17	85.23 ± 13.95 a	16.4	88 ± 12 ab	13.7
	Clone PD6	72.99 ± 14.12 a–e	19.3	80 ± 10 a–d	12.5
	TGR	63.56 ± 14.74 d–e	23.2	76 ± 12 b–d	15.3
	Clone 1	64.40 ± 12.04 c–e	18.7	84 ± 13 a–c	15.7
	Clone 3	73.99 ± 13.18 a–e	17.8	81 ± 10 a–d	12.7
	TDG	73.00 ± 15.91 a–c	21.8	81 ± 10 a–d	12.1
	Clone TOMBESI	82.68 ± 13.60 a–d	16.4	91 ± 5 a	5.2
Average (2020/21)	TGDL	67.74 ± 12.81 CD	18.9	71 ± 3 C	3.8
	Clone MT4	81.61 ± 12.46 A–C	15.3	79 ± 9 A–C	11.0
	Clone MT5	81.13 ± 8.34 A–C	10.3	80 ± 8 A–C	9.8
	Clone AD17	85.52 ± 6.75 A	7.9	86 ± 11 AB	12.4
	Clone PD6	71.20 ± 3.41 B–D	4.8	80 ± 6 A–C	8.0
	TGR	64.53 ± 13.13 D	20.4	76 ± 11 BC	14.1
	Clone 1	70.63 ± 9.48 B–D	13.4	80 ± 7 A–C	8.3
	Clone 3	73.97 ± 4.57 A–D	6.2	81 ± 7 A–C	8.8
	TDG	75.62 ± 13.23 A–D	17.5	80 ± 1 A–C	1.6
	Clone TOMBESI	83.39 ± 6.97 AB	8.4	88 ± 2 A	2.7
Average (2020)		77.26 ± 13.79 A	17.9	78 ± 9 A	11.4
Average (2021)		73.81 ± 14.68 A	19.9	82 ± 11 A	13.3
Overall Mean		75.53	–	80	–

* *Mean value ± Standard deviation (SD). Means followed by different letter(s) are significantly different at P ≤ 0.05 according to Fisher’s least significant difference (LSD) test*.

Variability of grafting success was also examined. As shown in Table 3, higher values were recorded for cultivars relative to their clones in both study years. In 2020, the coefficient of variation (CV) in cultivars ranged from 19.5% (TDG) to 22.5% (TGR), while in their clones it ranged from 15.6% (C1) to 18.5% (PD6). In 2021, the CV values ranged from 21.8% (TDG) to 23.2% (TGR) for cultivars and from 16.4% (Tombesi and AD17) to 19.3% (PD6) for clones.

### The Percentage of Class I Grafted Plants

The percentage of Class I grafted plants produced in 2020 ranged from 73% (TGDL cultivar) to 85% (Tombesi clone), with no significant differences between cultivars and their clones (Table 3). On the other hand, in 2021, significant differences in the production of Class I grafted plants were recorded, whereby significantly more Class I grafted plants were obtained from the Tombesi clone (91%) compared to the TGDL (69%) and TGR (76%) cultivars. In addition, significantly more Class I grafted plants were obtained from clones AD17, MT5 and Clone 1 compared to the cultivar TGR. There were no significant differences between the remaining cultivars and clones, where the percentage of Class I grafted plants ranged from 76% (TGR) to 81% (TDG and clones Clone 2, MT4 and PD6).

When the data for both examined years were analyzed jointly (Table 3), the findings revealed that the best grafting success was achieved by clone AD17 (85.52%), which is significantly higher than the values obtained for cultivars TGR (64.53%) and TGDL (67.74%), as well as Clone 1 (70.63%) and PD6 (71.20%). The coefficient of variation for the average grafting success for the two-year study period ranged from 17.5% (TDG) to 20.4% (TGR) in cultivars and was significantly lower in clones, spanning from only 6.2% (Clone 3) to 15.3% (clone MT4). Therefore, a much more stable grafting success was observed in clones in relation to their basic cultivars. Considering the two-year average, all clones had a higher grafting success in relation to their basic cultivars. However, the percentage success of graft acceptance was not significant in all cases. Therefore, these differences were primarily reflected in the uniformity (stability) of grafting success through lower clone correlation coefficient values.

As can be seen from Table 3, the average Class I grafted plant production success for the examined two-year period was 80%, with the majority of Class I grafted plants obtained by grafting clones Tombesi (88%) and AD17 (86%), while a significantly lower share of Class I grafted plants was obtained from the basic cultivars TGDL and TGR (71 and 76%, respectively).

### The Height and Diameter of Grafted Plants

The ANOVA results related to the tree height variance (Table 4) indicated a significant effect of Cultivars and their Clones (C) on the overall height variability among grafted plants, while the effect of year (Y) was not statistically significant. Both analyzed factors (Y and C) exerted a highly significant influence (Pr < 0.0001) on the graft union diameter, whereas the influence of the C × Y interaction on any of the examined traits was not significant.

**Table 4:** ANOVA results related to the height and diameter of grafted plants.^*^

Source	d.f.	Plant Height			Plant Diameter		
		MS	*F*	Pr > *F*	MS	*F*	Pr > *F*
Model	19	147.075	1.198	0.290	22.853	4.633	< 0.0001
Y	1	380.772	3.101	0.083	88.200	17.883	< 0.0001
C	9	268.110	2.183	0.036	34.689	7.033	< 0.0001
Y × C	9	0.074	0.001	1.000	3.756	0.761	0.652
Error	60	122.802	–	–	4.932	–	–
Total	79	–	–	–	–	–	–

* *ANOVA, Analysis of variance involving basic parameters: d.f., Degrees of freedom; MS, Mean squares; F, Variance ratio (F test); Pr, Probability value corresponding to a variance ratio*.

The results of measuring grafted plant height (cm) and diameter (mm) are shown in Table 5. In both analyzed years, the greatest grafted plant height was achieved in clones AD17 and Tombesi. The height of clone AD17 in 2021 (189 cm) was significantly greater relative to TGR in both years, TGDL in 2020, and clones PD6, MT5, C3, and MT4 in 2020. In addition, the height of clone AD17 in 2020 and clone Tombesi in both years was greater than that achieved with the remaining clones and cultivars, but this difference was not statistically significant according to Duncan’s multiple range test. Although ANOVA did not indicate a significant impact of the year on the height variability among grafted plants, the results reported in Table 5 show that the average height in 2021 was 5 cm greater than that measured in 2020. The clone AD17 had the highest grafted plant height (187 cm) across both years, but the increase relative to the Tombesi clone (180 cm) was not statistically significant. The grafted plant height of clone AD17 was statistically significantly greater compared to all other cultivars and clones, while the height of Tombesi clone did not differ significantly from other cultivars and clones.

**Table 5:** The height (cm) and diameter (mm) of grafted plants within two years (Y), and ten cultivars and their clones (C), and the corresponding coefficient of variation (CV)^*^.

Year	Cultivars/Clones	Plant Height (cm)	CV (%)	Plant Diameter (mm)	CV (%)
2020	TGDL	168 ± 10 b	6	19 ± 2 de	13
	Clone MT4	170 ± 9 b	5	17 ± 3 e	16
	Clone MT5	168 ± 11 b	6	21 ± 2 b–d	10
	Clone AD17	184 ± 10 ab	5	24 ± 2 ab	7
	Clone PD6	167 ± 15 b	9	20 ± 3 c–e	14
	TGR	166 ± 13 b	8	19 ± 2 de	10
	Clone 1	173 ± 11 ab	6	23 ± 2 a–c	9
	Clone 3	169 ± 10 b	6	22 ± 2 b–d	8
	TDG	172 ± 11 ab	6	22 ± 2 b–d	10
	Clone TOMBESI	177 ± 9 ab	5	24 ± 2 ab	8
2021	TGDL	172 ± 10 ab	6	20 ± 3 c–e	13
	Clone MT4	174 ± 10 ab	5	22 ± 3 b–d	13
	Clone MT5	172 ± 11 ab	6	23 ± 2 a–c	9
	Clone AD17	189 ± 10 a	5	27 ± 2 a	6
	Clone PD6	171 ± 15 ab	9	22 ± 3 b–d	13
	TGR	170 ± 13 b	8	22 ± 2 b–d	9
	Clone 1	177 ± 11 ab	6	23 ± 2 a–c	10
	Clone 3	173 ± 10 ab	6	23 ± 2 a–c	8
	TDG	176 ± 11 ab	6	24 ± 2 ab	9
	Clone TOMBESI	182 ± 10 ab	5	26 ± 2 a	8
Average (2020/21)	TGDL	170 ± 9 B	5	20 ± 2 D	9
	Clone MT4	172 ± 9 B	5	20 ± 2 D	13
	Clone MT5	170 ± 11 B	6	22 ± 2C	8
	Clone AD17	187 ± 10 A	5	26 ± 1 A	5
	Clone PD6	169 ± 15 B	9	21 ± 1 CD	7
	TGR	168 ± 13 B	8	21 ± 1 CD	5
	Clone 1	175 ± 11 B	6	23 ± 2 BC	9
	Clone 3	171 ± 10 B	6	23 ± 2 BC	7
	TDG	174 ± 11 AB	6	23 ± 1 BC	5
	Clone TOMBESI	180 ± 10 AB	5	25 ± 1 AB	4
Average (2020)		171 ± 11 A	6	21 ± 3 B	14
Average (2021)		176 ± 11 A	6	23 ± 3 A	12
Overall Mean		173	–	22	–

* *Mean value ± Standard deviation (SD). Means followed by different letter(s) are significantly different at P ≤ 0.05 according to Duncan’s multiple range test*.

In both years, clones AD17 and Tombesi had the largest grafted plant diameter (Table 5), while this effect was noted for the cultivar TDG in 2021 only (ranging from 24 mm to 27 mm). These cultivars and clones had a significantly larger diameter compared to the TGDL cultivar in both years, as well as the TGR cultivar and clones PD6 and MT4 in 2020 (ranging from 17 mm to 20 mm). Based on a two-year average, the largest diameter was measured for the clone AD17 (26 mm), followed by the Tombesi clone (25 mm), and these diameters were statistically significantly greater than those measured for the remaining cultivars and clones, except for cultivar TGD and clone C1 (23 mm in both cases). Statistically significantly the smallest diameter was measured for the TGDL cultivar and MT4 clone (20 mm in both cases) in relation to all other cultivars and clones, except cultivar TGR and clone PD6.

## Discussion

Traditionally, hazelnuts have been propagated on their own roots (Tous et al., 2009) by suckers and layering, but this method typically resulted in a very low propagation rate and a longer juvenility period. To overcome these issues, other methods of propagation have been developed, such as grafting, cuttings, and micropropagation (Olsen and Smith, 2013), yielding good outcomes in both experimental trials and practice (Tous et al., 2009; Ellena et al., 2014). Grafting is used for a variety of reasons, including better control of vegetative propagation, reducing the time to full productivity, and increasing tolerance to biotic or abiotic stresses (Mudge et al., 2009).

Unlike most other fruit species, hazelnut is commonly propagated on its own roots due to the ease of propagation through rooted suckers. However, this often leads to sucker production at the base of the trunk, which must be regularly controlled, with adverse effects on the production cost, environment (when using herbicides) and disease spread (through the cut surface). The use of *C. colurna* L., a species native to the Balkans and Caucasus, as a rootstock can eliminate these issues (Korać et al., 1997a,b; Wilkinson, 2005), while improving drought and frost resistance (Hartmann et al., 1990; Janick and Paull, 2008; Ninić-Todorović et al., 2008) and increasing the nut and kernel size (Miletić et al., 2008). These benefits are evidenced by grafted hazelnut trees in Serbia that are still in prime condition and exhibit good productivity (Cerović et al., 2020) despite being grafted more than 50 years ago (Korać and Slović, 1973).

There are morphological differences in root systems between vegetative and seedling rootstocks. Vegetative rootstocks produce fewer primary roots, often no taproot and have a shallower root system (Hartmann et al., 1990). Seedling rootstocks of *C. colurna* L. produce deep-rooted trees that do not blow over in windstorms that would topple the shallow-rooted *C. avellana* (Janick and Moore, 1996). The deep-rooted trees are also more resistant to drought and are suited for non-irrigated orchards.

One of the main indicators of the applicability of propagation methods is grafting success. Grafting success depends on the intrinsic factors, such as compatibility and polarity, as well as some extrinsic factors, such as the alignment of the contact area, pressure and tissue adhesion, temperature, and humidity around the graft point (Loreti et al., 2019). According to Tedesco et al. (2020), the graft success of grape vine is also dependent on the rooting ability of the rootstock. While genetic control of grafting compatibility was reported with *Prunus* by Pina et al. (2021), such control has not been reported with *Corylus*. Hazelnut grafting has been studied by several authors who have obtained variable results depending on the grafting method, plant age, the time of year, and the location (Rodriguez et al., 1989). Some earlier studies revealed that hazelnut grafting is quite difficult due to very slow callus formation (Tombesi, 1985). As warm temperatures at the graft union increase grafting success (Lagerstedt, 1981), this finding has renewed interest in the use of vegetative rootstocks for hazelnut (Janick and Moore, 1996; Tous et al., 2009). Several authors have reported on hazelnuts grafted on Turkish filbert (Bush, 1941; Gellatly, 1956; Lagerstedt and Byers, 1969; Lagerstedt, 1971; Ayfer et al., 1986; and in Serbia Korać et al., 1995, 1996a, 1996c). According to Lagerstedt (1971), the first record of hazelnut grafting dates back to 1841, indicating that it began much later relative to other fruit species in which grafting has been used for thousands of years. The author further points out that cultivars grafted on Turkish filbert behave differently and states that the poorer reception of hazel grafts grafted on Turkish filbert is not caused by poor compatibility, but is rather due to the shortcomings in the grafting technique used at the time.

More recently, Rahemi et al. (2016) observed that the hypocotyl cleft graft is a simple method to increase the success rate of hazelnut grafting when the scion is coated with a thin layer of paraffin (wax). In their study, Hazelnut cultivar Carmela (*Corylus avellana*) was grafted on etiolated hypocotyls of native hazelnuts (*C. americana*). Uncoated scions had a 9% success rate, which increased to 85% when the scion was coated with a thin layer of paraffin immediately after grafting. In our research, the whip and tongue grafting method (which was applied between the end of March and the end of April) resulted in a two-year average grafting success of 75.53% for all cultivars and clones. The greatest success in both study years (85.80% in 2020 and 85.23% in 2021) was achieved by clone AD17 (clone of the cultivar TGDL). Achim et al. (2001) obtained a 62.7–68.7% success rate by grafting in June using the chip-budding method, noting that grafting through the whip and tongue method during winter was also successful.

Therefore, the technology described in this work (which was initially developed in Serbia) is commercially viable, as confirmed by previous research (Cerović et al., 2007, 2008, 2020). Based on a comparative study of the behavior of planting material of three hazelnut cultivars (Tonda di Giffoni, Tonda Gentille delle Langhe, and Tonda Romana) obtained by grafting on *C. colurna* and rooting of shoots, Carvajal Rodriguez et al. (2018) stated that the use of grafting for hazelnut propagation could reduce the unproductive period and decrease plant vigor, thus shifting carbohydrate partitioning from vegetative to reproductive activity.

In our study, year and year-by-cultivar/clone interactions didv not exhibit a significant effect on the total variability in the grafting success. This was a surprising finding, given the large differences in temperature, precipitation, and Rh between the years (Figures 1, 2), all of which are factors known to influence the grafting success of other plants (Loreti et al., 2019). However, in both examined years, the variability was greater in cultivars compared to their clones, suggesting that clones derived from their basic cultivars had greater uniformity (i.e., more stable grafting success). Similar findings were reported by Monastra et al. (1997), who noted the existence of minor variations among clones selected from the TGR population, while Valentini et al. (2001) found significant differences between clones obtained from the Tonda Gentile delle Langhe population. These results are in accordance with those yielded by recent research conducted by Oztolan-Erol et al. (2021) who identified high genetic diversity within the cultivar itself. The obtained results further indicate that all clones had higher grafting success compared to their basic cultivars. The only exception was clone PD6 (in 2020 only) which had lower grafting success compared to the basic cultivar TGDL, but the difference was not statistically significant.

Class I hazelnut grafted plants comprised 80% of the average two-year production output, most of which was obtained by grafting clones Tombesi (88%) and AD17 (86%), followed by basic cultivars TGDL and TGR (at 71 and 76%, respectively). Considering that only 30–50% Class I grafted plants are typically obtained in walnut production in Serbia (Bogdanović et al., 2019)., this is a considerable achievement.

By improving the hazelnut planting material production technology by grafting on Turkish filbert seedlings, as well as with cultivar selection and breeding programs, and selection of new promising clones with improved pomological characteristics, some of the most important goals in improving hazelnut production technology have been met (Fideghelli and De Salvador, 2009). The morphological, physical and chemical characteristics of the hazelnut nut depend on the genotype and its interaction with the environment, including the way hazelnuts are stored (Bignami et al., 1999; Ozdemir et al., 2001). For this reason, the continuous study of phenological and pomological traits can clarify the relationship between genotype and environmental factors, which would be beneficial to breeders, producers, and the food industry (Cristofori et al., 2007, 2008).

## Conclusion

The obtained results revealed that the chosen hazel cultivars and clones exhibited excellent grafting success rate when grafted on non-clonal *C. colurna* rootstocks. In both analyzed years, as well as throughout the entire study period, greater grafting success was achieved using clones relative to the basic cultivars. Over the two-year study period, the highest grafting success was achieved by clone AD17 (85.52%), and it was significantly higher in relation to cultivars TGR and TGDL (64.53 and 67.74%), as well as clones Clone 1 and PD6 (70.63 and 71.20%). The two-year average share of Class I grafted plants was 80%, arising primarily from grafting Tombesi clones (88%) and AD17 (86%), while a significantly lower share of Class I grafted plants was obtained using basic cultivars TGDL and TGR (71 and 76%, respectively). Apart from the aforementioned advantages, clones AD17 and Tombesi produced grafted plants of the greatest height and graft union diameter.

Variability of grafting success was also higher for cultivars relative to their clones in both study years while the year itself did not affect the grafting success. Thus, it can be concluded that clones, in addition to higher seedling acceptance, also had greater uniformity, that is, more stable grafting success in relation to the basic cultivars from which they were obtained. After adequate care, hazelnut grafted plants produced according to the technology developed at the Faculty of Agriculture in Novi Sad were ready for planting in the fall of the same year in which they were grafted. Together with the production of rootstocks, the production of grafted hazel grafted plants lasts a total of three growing seasons. From all the information presented above, it can be concluded that the technology involving grafting cultivars and clones of *C. avellana* L. on the *C. colurna* L. non-clonal rootstock established in Serbia is highly suitable for commercial production of quality hazelnut planting material grown in the form of a single tree.

## Data Availability Statement

The raw data supporting the conclusions of this article will be made available by the authors, without undue reservation.

## Ethics Statement

Written informed consent was obtained from the individual(s) for the publication of any potentially identifiable images or data included in this article.

## Author Contributions

SB and NM were the principal investigators. SB, SD, and GJ contributed to conception and design of the study. SD and DJ contributed and organized the grafting. SD and BB organized the data collection and the database. GJ performed the statistical analysis and wrote sections of the manuscript. NM contributed to writing the introduction of the manuscript. All authors contributed to manuscript revision, read, and approved the submitted version.

## Conflict of Interest

The authors declare that the research was conducted in the absence of any commercial or financial relationships that could be construed as a potential conflict of interest.

## Publisher’s Note

All claims expressed in this article are solely those of the authors and do not necessarily represent those of their affiliated organizations, or those of the publisher, the editors and the reviewers. Any product that may be evaluated in this article, or claim that may be made by its manufacturer, is not guaranteed or endorsed by the publisher.
